# It’s not always about me: The effects of prior beliefs and stimulus prevalence on self–other prioritisation

**DOI:** 10.1177/1747021820913016

**Published:** 2020-04-15

**Authors:** Johanna K Falbén, Marius Golubickis, Darja Wischerath, Dimitra Tsamadi, Linn M Persson, Siobhan Caughey, Saga L Svensson, C Neil Macrae

**Affiliations:** 1 School of Psychology, University of Aberdeen, Aberdeen, UK; 2 School of Psychology, University of Plymouth, Plymouth, UK

**Keywords:** Self-prioritisation, prior beliefs, ownership, stimulus prevalence, response bias, decision-making

## Abstract

Although self-relevance is widely acknowledged to enhance stimulus processing, the exclusivity of this effect remains open to question. In particular, in commonly adopted experimental paradigms, the prioritisation of self-relevant (vs. other-relevant) material may reflect the operation of a task-specific strategy rather than an obligatory facet of social-cognitive functioning. By changing basic aspects of the decisional context, it may therefore be possible to generate stimulus-prioritisation effects for targets other than the self. Based on the demonstration that ownership facilitates object categorisation (i.e., self-ownership effect), here we showed that stimulus prioritisation is sensitive to prior expectations about the prevalence of forthcoming objects (owned-by-self vs. owned-by-friend) and whether these beliefs are supported during the task. Under conditions of stimulus uncertainty (i.e., no prior beliefs), replicating previous research, objects were classified more rapidly when owned-by-self compared with owned-by-friend (Experiment 1). When, however, the frequency of stimulus presentation either confirmed (Experiment 2) or disconfirmed (Experiment 3) prior expectations, stimulus prioritisation was observed for the most prevalent objects regardless of their owner. A hierarchical drift diffusion model (HDDM) analysis further revealed that decisional bias was underpinned by differences in the evidential requirements of response generation. These findings underscore the flexibility of ownership effects (i.e., stimulus prioritisation) during object processing.

For over four decades, research has revealed the benefits of self-relevance during stimulus processing. Compared with material paired with other people (e.g., friend, mother, celebrity), information associated with the self is easier to detect, evaluate, and remember (e.g., Bargh & Pratto, 1986; Keyes & Brady, 2010; Kuiper & Rogers, 1979; Shapiro et al., 1997; Symons & Johnson, 1997). Underlining the potency of this effect, Sui et al. (2012) recently demonstrated that the advantages of self-relevance extend even to trivial stimuli. After coupling geometric shapes with various person labels (e.g., circle = you, triangle = best friend, square = stranger), participants’ perceptual-matching judgements (i.e., do shape–label stimulus pairs match the learned associations?) were fastest and most accurate for stimulus pairs associated with the self (vs. best friend or stranger), a phenomenon dubbed the *self-prioritisation effect* (Janczyk et al., 2019; Sui et al., 2012, 2014, 2015; Sui & Humphreys, 2017). Driving this effect, it has been argued, is a mind that is preferentially tuned to personally meaningful information, such that—via bottom-up attentional capture—self-relevance enhances stimulus processing (Humphreys & Sui, 2016; Sui & Humphreys, 2015, 2017; Sui & Rotshtein, 2019).

Beyond shape–label associations, self-prioritisation effects have been demonstrated across different experimental paradigms, stimuli, and sensory modalities (Frings & Wentura, 2014; Mattan et al., 2015; Moradi et al., 2015; Payne et al., 2017; Schäfer et al., 2015; Wozniak & Knoblich, 2019). In particular, object ownership has proved a productive task context for exploring the process and products of self-referential mentation (e.g., Constable et al., 2011, 2014, 2019; Cunningham et al., 2008; Falbén et al., 2019; Golubickis et al., 2018, 2019, in press; Lockwood et al., 2018; Sparks et al., 2016; Truong et al., 2017; Turk et al., 2011). For example, Golubickis et al. (2018) presented participants with objects (i.e., pencils and pens) that ostensibly belonged either to the self or a best friend, and their task was simply to classify the items (i.e., owned-by-self vs. owned-by-friend) as quickly as possible. The results yielded a self-prioritisation effect, revealing response facilitation for self-owned compared with friend-owned objects. Relatedly, in a task probing the temporal order of stimulus presentation (i.e., prior-entry effect), Constable et al. (2019) demonstrated that self-owned objects were reported to appear first more frequently than comparable items that belonged to the experimenter. Taken together, these findings underscore the influence that ownership exerts during stimulus processing (Beggan, 1992; Kahneman et al., 1991; Morewedge & Giblin, 2015; Pierce et al., 2003). Questions remain, however, regarding the exclusivity of this self-ownership effect. Specifically, is stimulus prioritisation restricted to objects owned by the self or can it extend to other people’s possessions?

## Ownership and decisional processing

The putative exclusivity of the self-ownership effect derives, at least in part, from its underlying origin. Despite the contention that self-relevance facilitates perceptual processing (Humphreys & Sui, 2016; Sui & Humphreys, 2015, 2017; Sui & Rotshtein, 2019; but see Reuther & Chakravarthi, 2017), studies manipulating object ownership have garnered little support for this viewpoint. Instead, self-prioritisation has been traced to the operation of a different underlying mechanism—a response bias (Constable et al., 2019). For example, using drift diffusion modelling to identify the processes supporting task performance (Ratcliff et al., 2016; Voss et al., 2013; White & Poldrack, 2014), Golubickis et al. (2018, 2019) demonstrated that the self-ownership effect was underpinned by variability in the evidential requirements of response generation, such that less information was needed to generate owned-by-self compared with owned-by-other decisions. Inter-estingly, no differences in the efficiency of stimulus processing were observed as a function of ownership. Together with related research, this reveals that, rather than enhancing stimulus salience, self-relevance expedites performance through its influence on post-perceptual (i.e., decisional) processing operations (Miyakoshi et al., 2007; Reuther & Chakravarthi, 2017; Siebold et al., 2015; Stein et al., 2016; Wade & Vickery, 2018).

According to Golubickis et al. (2018), reduction in the evidential requirements of decision-making reflects the operation of an egocentric response-related strategy. Specifically, people display an a priori preference for self-related compared with other-related responses. Although egocentrism is most strongly associated with childhood (Perner, 1991; Wimmer & Perner, 1983), adults continue to behave in distinctly self-centric ways. Indeed, it has been suggested that all that separates children from their elders are corrective processes that flexibly counteract the effects of egocentrism. In other words, it is not that adults are less self-centred than children; rather, they are simply better able to suppress and modify their preliminary egocentric reactions (Epley et al., 2004; Epley & Gilovich, 2004). Crucially, egocentrism and ownership are closely intertwined (Cunningham et al., 2013). From around the age of 18 to 24 months, children begin to use possessive pronouns (e.g., mine) and, by the age of 4, have a concrete understanding of ownership (Hay, 2006). Indeed, conflict between siblings and peers routinely derives from disputes over proprietorship (Furby, 1980; Ramsey, 1987). In Golubickis et al.’s (2018, 2019) object-classification task, a response bias is indicative of the operation of an egocentric task-related strategy. That is, repeated interactions with one’s own (vs. other people’s) possessions create a preference for self-relevant responses (i.e., prior experience tunes decisional processing).

## Self-bias under conditions of uncertainty

Although providing a viable explanation for the self-ownership effect, an egocentric response-related strategy may reflect the operation of a transitory task-related tactic rather than an inevitable facet of decisional processing. Inspection of the paradigm employed by Golubickis et al. (2018, 2019) suggests why. In their experiment, prior to the object-classification task, pencils or pens were randomly assigned to the self and a best friend. Critically, no other task-relevant information was provided. In particular, the composition of the sample of objects (i.e., the number of pencils and pens) was unspecified. Under such conditions of stimulus uncertainty, it is possible that object relevance served as the most salient dimension of the task, thereby triggering a self-prioritisation effect that was grounded in an egocentric response-related bias (Epley & Gilovich, 2004). In other words, self-bias materialised as a strategic response to the prevailing task conditions (i.e., stimulus uncertainty), rather than as an obligatory product of social-cognitive functioning (Constable et al., 2019; Falbén et al., 2019; Golubickis et al., 2018, 2019). Had additional task-relevant details been available—specifically information about the prevalence of the to-be-judged items—then object processing (hence stimulus prioritisation) may have taken an entirely different course.

Outside the laboratory, judgements of ownership unfold in informationally rich settings. Consider, for example, two dwellings: one’s own and a close friend’s apartment. Whereas the items in one’s home are principally owned by the self, this is patently not the case at a friend’s place where perhaps only a small collection of one’s belongings may reside. Would it therefore make sense to prioritise self-owned (i.e., infrequent) compared with friend-owned (i.e., frequent) objects when judging the ownership of items sampled from the latter setting? We suspect not. Rather, processing would be optimised if individuals were sensitive to the likelihood of encountering (self-owned vs. other-owned) objects in contexts in which their occurrence varies. That is, decision-making is guided by the extent to which prior beliefs about the world are consistent with the available sensory data (Bar, 2007). Supporting this viewpoint is the demonstration that low-probability stimuli are detected less rapidly than their high-probability counterparts (Hon et al., 2013; Wolfe et al., 2007). Generally speaking, processing is facilitated for probable compared with improbable items, with prior expectations about the likely appearance of stimuli shaping the cognitive operations that underpin decisional processing (De Loof et al., 2016; Dunovan et al., 2014).

Of course, despite the obvious utility of information signalling the prior probability of an event or outcome, an extensive literature has demonstrated that people can be notoriously unreceptive to this knowledge, an effect termed base-rate neglect (e.g., Barbey & Sloman, 2007; Tversky & Kahneman, 1974). For example, confronted with reams of statistical information about the reliability of German automobiles, a potential purchaser may nonetheless be persuaded not to buy one on learning that a colleague’s BMW broke down on a recent vacation. That is, diagnostic information is ignored in lieu of a seemingly compelling single experience. As it turns out, however, overlooking base-rates in this way largely occurs when the decisional value of the information is uncertain. When probabilistic information is plainly pertinent (e.g., causally relevant) to the judgement at hand, it is routinely taken into consideration during decision-making (Ajzen, 1977; Bar-Hillel, 1980; Ginosar & Trope, 1980; Pennycook & Thompson, 2012). This observation has direct implications for the generation of ownership effects during object processing (Constable et al., 2019; Golubickis et al., 2018). If egocentric responses reflect the adoption of a task-specific strategy, then self-prioritisation should emerge under conditions of uncertainty when the likelihood of encountering self-owned or other-owned objects is unknown. When, in contrast, this information is available (i.e., self-owned or other-owned objects are known to predominate), prioritisation effects should arise regardless of who possesses the items.

## The current research

Extending previous research, here we considered whether prior beliefs pertaining to the likelihood of encountering either self-owned or friend-owned material during an object-classification task influences stimulus prioritisation. Following Golubickis et al. (2018), a hierarchical drift diffusion model (HDDM) analysis was conducted to identify the processes underpinning task performance (Wiecki et al., 2013). The drift diffusion model uses both accuracy and response latency to represent the decision-making process as it unfolds over time, thereby enabling the latent cognitive operations associated with task performance to be estimated (Ratcliff et al., 2016). During binary decision-making (e.g., is an object owned-by-self or owned-by-friend?), information is continuously accumulated from a stimulus until sufficient evidence is acquired to make a response. The advantage of this analytic approach resides in the ability of the HDDM to distinguish between biases in stimulus and response-related processes. In the drift diffusion framework, these biases are conceptually distinct, with different underlying origins and theoretical interpretations (Ratcliff et al., 2016; Voss et al., 2013; White & Poldrack, 2014).

The drift rate (*v*) estimates the speed and quality of information acquisition (i.e., larger drift rate = faster information uptake), thus is interpreted as a measure of the efficiency of stimulus processing during decision-making. For example, during stimulus appraisal, self-relevance may facilitate information uptake for self-owned compared with friend-owned objects, thereby demonstrating that self-prioritisation is underpinned by a stimulus bias (Humphreys & Sui, 2016; Sui & Humphreys, 2015, 2017; Sui & Rotshtein, 2019). Boundary separation (*a*) estimates the distance between the two response thresholds (i.e., how much information is required before a decision is made), and the starting point (*z*) specifies the position between the response thresholds at which evidence accumulation begins. If *z* is not centred between the thresholds, this indicates a bias in favour of the response that is closer to the starting point (i.e., less evidence is required to reach the preferred threshold). For example, self-relevance may modulate information-sampling requirements, such that less evidence is needed to generate owned-by-self than owned-by-friend responses, indicating that self-prioritisation is underpinned by a response bias (White & Poldrack, 2014).

As previously noted, under conditions of stimulus uncertainty (i.e., no prior beliefs about the prevalence of self-owned vs. friend-owned objects), the self-ownership effect was underpinned by a response bias (Golubickis et al., 2018, 2019). Specifically, self-prioritisation corresponded to a shift in the starting point of evidence accumulation (*z*), such that less information was required to generate owned-by-self compared with owned-by-other (e.g., friend or mother) responses. Response biases commonly arise when one outcome is more likely than another, resulting in a higher starting value for the probable (vs. improbable) response (De Loof et al., 2016; Domenech & Dreher, 2010; Dunovan et al., 2014; Mulder et al., 2012; White & Poldrack, 2014). If therefore participants are sensitive to information indicating the likelihood of encountering either self-owned or friend-owned items during an object-classification task, then it should be possible, at least in principle, to elicit both self- and friend-ownership effects, with each effect underpinned by differences in the respective evidential requirements of response generation.

## Experiment 1

The goal of Experiment 1 was to establish the extent to which self-prioritisation is sensitive to prior information indicating the likelihood that self-owned (vs. friend-owned) items will be encountered during an object-classification task. Following Golubickis et al. (2018, 2019), participants were presented with objects (i.e., pencils and pens) that ostensibly belonged either to the self or a friend and their task was simply to classify the items as a function of their ownership. Critically, whereas half the participants were given no prior information about the sample of objects (i.e., “stimulus-uncertainty” condition), the others were told there was an equal likelihood (i.e., “equal-probability” condition) of encountering pencils and pens (i.e., self-owned vs. friend-owned objects) during the task. We hypothesised that a self-ownership effect would emerge only under conditions of stimulus uncertainty (Golubickis et al., 2018, 2019). To identify the processes underpinning task performance, data were submitted to an HDDM analysis (Wiecki et al., 2013).

### Method

#### Participants and design

Seventy-two undergraduates (17 male, *M_age_* = 20.82, *SD* = 3.57) took part in the research.^1^ Two participants (male) failed to follow the instructions by responding with invalid key presses, thus were excluded from the analyses. All participants had normal or corrected-to-normal visual acuity. Informed consent was obtained from participants prior to the commencement of the experiment and the protocol was reviewed and approved by the Ethics Committee at the School of Psychology, University of Aberdeen, Scotland. The experiment had a 2 (Expectancy: none vs. equal) × 2 (Owner: self vs. friend) mixed design with repeated measures on the second factor.

#### Stimulus materials and procedure

Participants arrived at the laboratory individually, were greeted by the experimenter, seated in front of a desktop computer, and informed that the experiment comprised an object-classification task featuring two categories of objects: pencils and pens. Following Golubickis et al. (2018), participants were told that, prior to the commencement of the task, the computer had randomly assigned one category of objects to them (i.e., owned-by-self) and the other category of objects to their best friend (i.e., owned-by-friend). That is, participants owned all the items (i.e., pencils or pens) from one of the categories, and their best friend owned all the items from the other category. They then pressed the spacebar on the keyboard and text appeared revealing who had been assigned the pencils and pens, respectively (e.g., you = pencils, friend = pens). Assignment of the objects to self and friend was counterbalanced across the sample.

The experimenter then explained that, on the computer screen, participants would be presented with a series of pictures of individual pencils and pens and their task was simply to report (via a button press), as quickly and accurately as possible, whether the item belonged to them or to their friend. Prior to the commencement of the task, a pencil sketch of a box (containing pencils and pens) appeared on the screen, giving indicative information about the trial structure during the task (see Supplementary Material for the sketches used in the current experiments). Importantly, for half the participants, the box comprised 50% pens and 50% pencils, thereby indicating there would be an equivalent number of self-owned and friend-owned items presented during the task (i.e., equal-probability, 50/50 condition). For the other participants, in contrast, the box was closed, so no information was available about the frequency of the trials (i.e., stimulus-uncertainty condition). Responses were given using two buttons on the keyboard (i.e., N and M). Key–response mappings were counterbalanced across participants and the labels “mine” and “friend” were located above the relevant response buttons.

Each trial began with the presentation of a central fixation cross for 1,000 ms, followed by the picture of a pencil or pen for 100 ms. After each object was presented, the screen turned blank until participants reported the owner of the item (i.e., self or friend). Following each response, the fixation cross reappeared and the next trial commenced. The two categories of stimuli comprised photographs of 28 unique objects (14 pencils and 14 pens) that were taken from Google images and edited using Photoshop CS6, such that each pencil or pen was oriented obliquely from the left-bottom to the right-top corner (see Supplementary Material). Images were 140 × 140 pixels in size, greyscale, and matched for luminance. Participants initially performed 16 practice trials, followed by one block of 224 trials in which all stimuli occurred equally often in a random order, with 112 trials in each condition (i.e., self-owned trials vs. friend-owned trials). All that differed across the task was that half the participants (i.e., equal-probability condition) had prior knowledge about the frequency of the trials. On completion of the task, participants were debriefed, thanked, and dismissed.

### Results and discussion

#### Response time and accuracy

**Figure 1.**
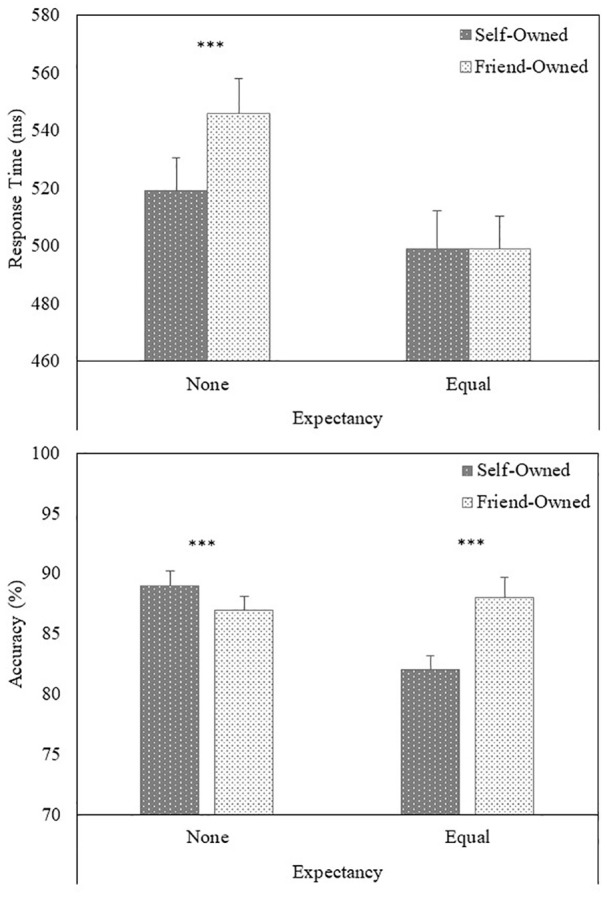
Task performance (upper panel = response time, lower panel = accuracy) as a function of Expectancy and Target (Experiment 1). Error bars represent +1 standard error of the mean Significant differences between conditions are depicted with asterisks: ***p < .001.

Responses faster than 200 ms were excluded from the analysis (Golubickis et al., 2018; Sui et al., 2012), eliminating less than 1% of the overall number of trials. As the response window was set to 2,000 ms, no outlier screening was performed for slow responses (Golubickis et al., 2018). A multilevel model analysis was used to examine the response time (RT) (i.e., correct responses) and accuracy data (see Figure 1). Analyses were conducted with the R package “lmer4” (Pinheiro et al., 2015), with Expectancy and Owner modelled as fixed effects and participants as a crossed random effect (Judd et al., 2012). Analysis of the RTs (correct responses) yielded a main effect of Owner (*b* = –.004, *SE* = .001, *t* = –3.25, *p* = .001), such that responses were faster to self-owned (*M* = 509 ms, *SD* = 97 ms) compared with friend-owned (*M* = 523 ms, *SD* = 103 ms) items. A significant Expectancy × Owner (*b* = .004, *SE* = .001, *t* = 2.75, *p* = .006) interaction also emerged. Further analysis of the interaction revealed that, when no expectancy was provided, responses were faster to self-owned compared with friend-owned objects (*b* = –.008, *SE* = .002, *t* = –4.36, *p* < .001). In contrast, no such effect emerged when participants were aware that equal numbers of self-owned and friend-owned objects would be presented during the task (an additional Bayesian analysis yielded moderate evidence for the null effect, *BF*_01_ = 5.43).

A multilevel logistic regression analysis on the accuracy of responses yielded a main effect of Owner (*b* = –.060, *SE* = .025, *z* = –2.40, *p* = .016), such that responses were more accurate for friend-owned (*M* = 88%, *SD* = 10%) than self-owned (*M* = 86%, *SD* = 12%) items. A significant Expectancy × Owner (*b* = –.185, *SE* = .025, *z* = –7.43, *p* < .001) interaction also emerged. Further analysis of the interaction revealed that, when no information about the frequency of trials was provided, responses were more accurate to self-owned compared with friend-owned objects (*b* = .125, *SE* = .036, *z* = 3.50, *p* < .001). This effect reversed when participants were aware that equal numbers of self-owned and friend-owned objects would be presented during the task (*b* = –.245, *SE* = .035, *z* = –7.07, *p* < .001).

#### Drift diffusion modelling

To identify the processes underpinning task performance, data were submitted to an HDDM analysis following the same outlier screening as the RT/accuracy analyses. HDDM is an open-source software package written in Python for the hierarchical Bayesian estimation of drift diffusion model parameters (Wiecki et al., 2013). This approach assumes that the model parameters for individual participants are random samples drawn from group-level distributions and uses Bayesian statistical methods to estimate all parameters at both the group and individual-participant level (Vandekerckhove et al., 2011). Models were response coded, such that the upper threshold corresponded to an “owned-by-self” response and the lower threshold to an “owned-by-friend” response (Golubickis et al., 2018, 2019).

Seven models were estimated for comparison (see Table 1). First, to investigate whether task performance was underpinned by differences in the efficiency of stimulus processing (i.e., stimulus bias), a model that allowed the drift rate (*v*) to vary as a function of Expectancy (i.e., no expectancy vs. equal expectancy) and Target (i.e., owned-by-self vs. owned-by-friend) was estimated. In the second model, starting point (*z*) was allowed to vary as a function of Expectancy. The third model allowed threshold separation (*a*) to vary across Expectancy and in the fourth model both starting point (*z*) and threshold separation (*a*) were allowed to vary as a function of Expectancy. These models (2–4) investigated whether task performance was underpinned by differences in information-sampling requirements (i.e., response bias). Finally, to examine whether task performance was underpinned by both stimulus and response biases, three models were estimated in which combinations of drift rate (*v*), starting point (*z*), and threshold separation (*a*) were allowed to vary. In all models, inter-trial variability was estimated for drift rate (*sv*), starting point (*sz*), and non-decision time (*st*).

**Table 1.** DIC for each model (Experiment 1).

Model	Expectancy	Owner	Fixed	DIC
1	*v*	*v*	*a, z*	−10,772
2	*z*	*–*	*a, v*	−10,898
3	*a*	*–*	*v, z*	−10,217
4	*a, z*	*–*	*v*	−10,218
5	*v, z*	*v*	*a*	−11,121
6	*a, v*	*v*	*z*	−10,772
7	*a, v, z*	*v*	*–*	−11,124

DIC: deviance information criterion; *v*: drift rate; *a*: threshold separation; *z*: starting point.

Bayesian posterior distributions were modelled using a Markov Chain Monte Carlo (MCMC) with 10,000 samples (with 1,000 burn in samples). As can be seen in Table 1, Model 7 yielded the best fit (i.e., lowest Deviance Information Criterion value, DIC). The DIC was adopted as it is routinely used for hierarchical Bayesian model comparison (Spiegelhalter et al., 1998). As diffusion models were fit hierarchically rather than individually for each participant, a single value was calculated for each model that reflected the overall fit to the data at the participant and group level. Lower DIC values favour models with the highest likelihood and least number of parameters. To further evaluate the best fitting model, a standard model comparison procedure used in Bayesian parameter estimation—Posterior Predictive Check (PPC)—was performed (Wiecki et al., 2013). For the best fitting model, the posterior distributions of the estimated parameters were used to simulate data sets. We then assessed the quality of model fit by plotting the observed data against the simulated data for the .1, .3, .5, .7, and .9 RT quantiles for each experimental condition (Krypotos et al., 2015). This revealed good model fit (see Supplementary Material for associated plots).

Inspection of the posterior distributions for the best fitting model indicated that task performance was underpinned by a combination of response and stimulus biases (see Table 2). When no expectancy was provided, comparison of the observed starting value (*z* = 0.53) with no bias (*z* = 0.50) yielded extremely strong evidence for a response bias, *p*_Bayes_(bias > .50) = .001,^2^ such that less information was needed to generate owned-by-self compared with owned-by-friend responses. There was no evidence for a starting point difference when self-owned and other-owned objects were equally likely to appear (*z* = 0.51). In addition, there was moderate evidence for a difference in threshold separation, *p*_Bayes_(none > equal) = .058, indicating that responses were more cautious when no expectancy (vs. equal) was provided. Finally, strong evidence for a stimulus bias was observed, revealing that information uptake was faster for friend-owned than self-owned objects, but only in the equal-probability condition, *p*_Bayes_(owned-by-friend > owned-by-self) = .006.

**Table 2.** Parameter means and the upper (97.5q) and lower (2.5q) quantiles of the best fitting model (Experiment 1).

Diffusion model parameter	Mean	Quantile	
		2.5q	97.5q
*a_none_*	1.144	1.037	1.258
*a_equal_*	1.027	0.919	1.141
*v_none/self-trial_*	3.108	2.686	3.543
*v_none/friend-trial_*	−3.061	−3.491	−2.651
*v_equal/self-trial_*	2.631	2.193	3.067
*v_equal/friend-trial_*	−3.395	−3.837	−2.960
*z_none_*	0.529	0.510	0.548
*z_equal_*	0.512	0.492	0.532
*t*_0_	0.347	0.333	0.362
*sv*	1.219	1.067	1.377
*sz*	0.569	0.515	0.620
*st*	0.212	0.207	0.217

*a*: threshold separation; *v*: drift rate; *z*: starting point; *t*_0_: non-decision time; *sv*: inter-trial variability in drift rate; *sz*: inter-trial variability in starting point; *st*: inter-trial variability in non-decision time.

Experiment 1 provided evidence that stimulus predictability moderates the emergence of the self-ownership effect during an object-classification task. Replicating Golubickis et al. (2018, 2019), under conditions of stimulus uncertainty (i.e., no trial-related information was available), compared with pencils or pens owned by a friend, identical self-owned items elicited faster responses. In contrast, self-prioritisation failed to emerge when participants were cognizant that self-owned and friend-owned objects were equally likely to appear. In other words, stimulus predictability eliminated the self-ownership effect. Also replicating Golubickis et al. (2018, 2019), under conditions of stimulus uncertainty, self-prioritisation was underpinned by a response bias (White & Poldrack, 2014). Specifically, less evidence was needed to generate owned-by-self compared with owned-by-friend responses. Taken together, these findings demonstrate that, at least in an object-ownership task, self-bias derives from the adoption of an egocentric decision-making strategy, but crucially only under conditions of stimulus uncertainty.

## Experiment 2

To date, only self-relevant objects have been reported to yield ownership effects (e.g., Constable et al., 2019; Golubickis et al., 2018; Truong et al., 2017). The results of Experiment 1, however, suggest that it should be possible to trigger comparable processing benefits for material that belongs to other individuals. Specifically, if people are forewarned about the likelihood of encountering self-owned or friend-owned objects then—when the latter objects predominate during the task (i.e., comprise the expected response)—a friend-prioritisation effect should emerge. That is, responses should be facilitated to friend-owned compared with self-owned items (cf. Golubickis et al., 2018, 2019). Thus, by providing prior information about which items are most likely to be encountered during the object-classification task (i.e., self-owned or friend-owned), it should be possible to generate both self- and friend-ownership effects. Extending Experiment 1, stimulus prevalence was manipulated within participants and, as previously, data were submitted to an HDDM analysis to identify the processes underpinning task performance (Wiecki et al., 2013).

### Method

#### Participants and design

Thirty-six undergraduates (seven male, *M_age_* = 20.19, *SD* = 1.67) took part in the research. All participants had normal or corrected-to-normal visual acuity. Informed consent was obtained from participants prior to the commencement of the experiment, and the protocol was reviewed and approved by the Ethics Committee at the School of Psychology, University of Aberdeen, Scotland. The experiment had a 3 (Expectancy: self-expected vs. friend-expected vs. equal) × 2 (Owner: self vs. friend) repeated-measures design.

#### Stimulus materials and procedure

Participants arrived at the laboratory individually, were greeted by the experimenter, seated in front of a desktop computer, and informed that the experiment comprised an object-classification task featuring two categories of objects: pencils and pens. The task closely followed Experiment 1, but with an important modification. In the current study, participants completed three blocks of trials in which the likelihood that self-owned and friend-owned objects would be presented was manipulated. Prior to the commencement of each block, a pencil sketch of a box (containing varying numbers of pencils and pens) appeared on the screen conveying diagnostic information about the frequency of trials during the block (e.g., Block 1, 75% pens and 25% pencils; Block 2, 25% pens and 75% pencils; Block 3, 50% pens and 50% pencils). The order of presentation of the blocks was counterbalanced across participants, with each block comprising 224 trials (e.g., Block 1, 168 self-trials and 56 friend-trials; Block 2, 56 self-trials and 168 friend-trials; Block 3, 112 self-trials and 112 friend-trials). On completion of the task, participants were debriefed, thanked, and dismissed.

### Results and discussion

#### RT and accuracy

**Figure 2.**
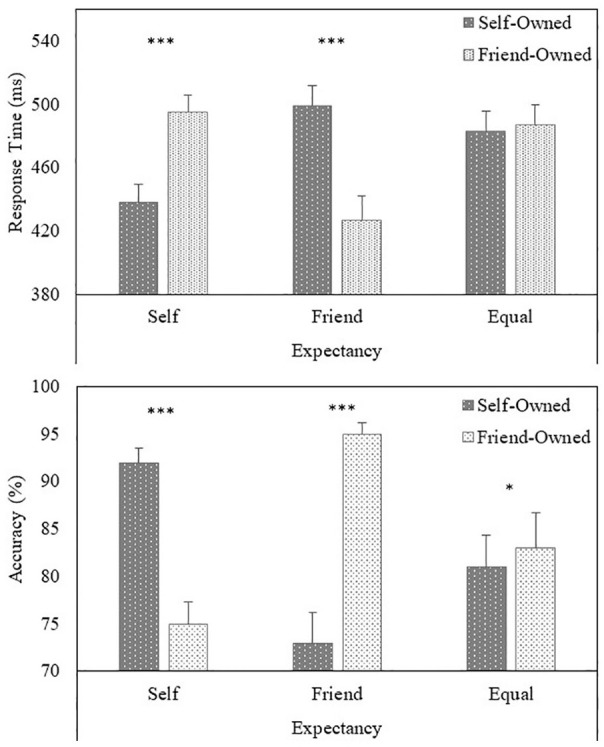
Task performance (upper panel = response time, lower panel = accuracy) as a function of Expectancy and Target (Experiment 2). Error bars represent +1 standard error of the mean Significant differences between conditions are depicted with asterisks: *p < .05. ***p < .001.

Responses faster than 200 ms were excluded from the analysis, eliminating less than 1% of the overall number of trials. A multilevel model analysis of the RTs (correct responses) yielded a main effect of Expectancy (*b* = .003, *SE* = .001, *t* = 2.27, *p* = .023), such that responses were faster for friend-owned (*M* = 470 ms, *SD* = 76 ms) than self-owned (*M* = 473 ms, *SD* = 75 ms) items. In addition, a significant Expectancy × Owner (*b* = –.037, *SE* = .001, *t* = –27.76, *p* < .001) interaction emerged (see Figure 2). Further analysis of the interaction revealed that, when self-trials were expected, responses were faster to self-owned compared with friend-owned objects (*b* = –.028, *SE* = .002, *t* = –14.64, *p* < .001). In contrast, when friend-trials were expected, responses were faster to friend-owned than to self-owned objects (*b* = .034, *SE* = .002, *t* = 16.09, *p* < .001). No difference emerged when self-trials and friend-trials were equally probable (an additional Bayesian analysis yielded moderate evidence for the null effect, *BF*_01_ = 4.69).

A multilevel logistic regression analysis on the accuracy of responses yielded a main effect of Owner (*b* = –.103, *SE* = .021, *z* = –5.04, *p* < .001), such that responses were more accurate to friend-owned (*M* = 84%, *SD* = 14%) than to self-owned (*M* = 82%, *SD* = 16%) items. In addition, a significant Expectancy × Owner (*b* = .912, *SE* = .027, *z* = 34.24, *p* < .001) interaction emerged (see Figure 2). Further analysis of the interaction revealed that, when self-trials were expected, responses were more accurate to self-owned compared with friend-owned objects (*b* = .771, *SE* = .038, *z* = 20.33, *p* < .001). In contrast, when friend-trials were expected, responses were more accurate to friend-owned than to self-owned objects (*b* = –1.035, *SE* = .041, *z* = –25.18, *p* < .001). When self- and friend-trials were equally likely, responses were more accurate to friend-owned compared with self-owned objects (*b* = –.084, *SE* = .034, *z* = –2.49, *p* = .013).

#### Drift diffusion modelling

As in Experiment 1, seven models in which combinations of drift rate (*v*) varying as a function of Expectancy (i.e., self-expected vs. friend-expected vs. equal) and Owner (i.e., self vs. friend), and starting point (*z*) and threshold separation (*a*) varying as a function of Expectancy were estimated for comparison. As can be seen from Table 3, Model 7 yielded the best fit (i.e., lowest DIC value). To further evaluate the model fit, a PPC was also performed. This revealed good model fit (see Supplementary Material for associated plots).

**Table 3.** DIC for each model (Experiment 2).

Model	Expectancy	Owner	Fixed	DIC
1	*v*	*v*	*a, z*	−20,922
2	*z*	–	*a, v*	−23,598
3	*a*	–	*v, z*	−19,209
4	*a, z*	–	*v*	−22,231
5	*v, z*	*v*	*a*	−23,046
6	*a, v*	*v*	*z*	−21,732
7	*a, v, z*	*v*	–	−24,957

DIC: deviance information criterion; *v*: drift rate; *a*: threshold separation; *z*: starting point.

Inspection of the posterior distributions for the best fitting model indicated that task performance was underpinned by a combination of response and stimulus biases (see Table 4). When self-trials were expected, comparison of the observed starting value (*z* = 0.62) with no bias (*z* = 0.50) revealed extremely strong evidence for a response bias, *p*_Bayes_(bias > .50) < .001, such that less information was needed to generate owned-by-self compared with owned-by-friend responses. Similarly, extremely strong evidence for a response bias was also observed when friend-trials were expected (*z* = 0.31), indicating that less information was required when generating owned-by-friend than owned-by-self responses, *p*_Bayes_(bias < .50) < .001. No evidence for a starting point difference emerged when self-trials and friend-trials were equally probable (*z* = 0.49). Also, no differences in threshold separation were observed. Finally, when friend-trials were expected, strong evidence for a stimulus bias was observed, *p*_Bayes_(owned-by-self > owned-by-friend) = .022, such that information uptake was faster for self-owned than friend-owned items.

**Table 4.** Parameter means and the upper (97.5q) and lower (2.5q) quantiles of the best fitting model (Experiment 2).

Diffusion model parameter	Mean	Quantile	
		2.5q	97.5q
*a_self-expected_*	1.133	1.045	1.217
*a_friend-expected_*	1.133	1.043	1.230
*a_equal_*	1.134	1.048	1.227
*v_self-expected/self-trial_*	2.401	2.015	2.788
*v_self-expected/friend-trial_*	*−*2.479	−2.875	−2.010
*v_friend-expected/self-trial_*	2.828	2.447	3.236
*v_friend-expected/friend-trial_*	*−*2.245	−2.636	−1.846
*v_equal/self-trial_*	1.989	1.590	2.356
*v_equal/friend-trial_*	*−*1.904	−2.289	−1.499
*z_self-expected_*	0.621	0.595	0.647
*z_friend-expected_*	0.309	0.284	0.334
*z_equal_*	0.494	0.466	0.522
*t*_0_	0.282	0.272	0.293
*sv*	0.131	0.006	0.299
*sz*	0.357	0.357	0.357
*st*	0.144	0.144	0.144

*a*: threshold separation; *v*: drift rate; *z*: starting point; *t*_0_: non-decision time; *sv*: inter-trial variability in drift rate; *sz*: inter-trial variability in starting point; *st*: inter-trial variability in non-decision time.

The results of Experiment 2 challenge the exclusivity of the self-ownership effect (Golubickis et al., 2018, 2019). As expected, stimulus prioritisation was sensitive to the likelihood of encountering self-owned and friend-owned items during an object-classification task. Specifically, whereas self-prioritisation was observed when self-owned items were expected, this switched to friend-prioritisation when friend-owned items comprised the predominant stimuli. Corroborating Experiment 1, when self-owned and friend-owned items were equally likely to appear, stimulus prioritisation was abolished. Underpinning the observed ownership effects were differences in the evidential requirements of response generation. Notably, less evidence was needed when responding to probable than improbable objects, regardless of their owner. These findings confirm that response biases arise when one outcome is more likely than another, such that the starting point of evidence accumulation is closer to the probable than improbable response threshold (De Loof et al., 2016; Domenech & Dreher, 2010; Dunovan et al., 2014; Mulder et al., 2012; White & Poldrack, 2014). Thus, depending on the prevalence of self-owned or friend-owned objects, it is possible to generate both self- and friend-ownership effects during decisional processing.

## Experiment 3

Experiment 2 revealed that stimulus prioritisation is moderated by the likelihood of encountering self-owned and friend-owned items during an object-classification task. Specifically, when either self-owned or friend-owned objects comprised the predominant stimuli, it was possible to trigger self- and friend-ownership effects, respectively. Based on previous research, a feature of the adopted paradigm was that knowledge of the task structure (i.e., likelihood of encountering pencils or pens during a particular block) served as a reliable predictor of the upcoming experimental trials (De Loof et al., 2016; Dunovan et al., 2014). That is, when participants were informed that the majority of trials would pertain to self-owned (or friend-owned) items (e.g., 75% self-trials and 25% friend-trials), this mapped directly onto the frequency with which the objects were presented during the task. This, of course, raises an interesting question. What would happen if prior expectancies were disconfirmed by the frequency of stimulus presentation during the task (i.e., prior beliefs are inaccurate)? For example, participants expected to encounter self-owned (or friend-owned) objects but, in reality, friend-owned (or self-owned) items were in the majority. Under conditions such as these, we suspect inaccurate prior expectancies (both self-related and friend-related) would be superseded by prioritisation effects based on the objects that predominated during the task (i.e., self-owned or friend-owned). That is, participants would optimise a probabilistic representation of the immediate stimulus environment, such that prioritisation (i.e., self or friend) would emerge for the most frequent items.

### Method

#### Participants and design

Thirty-six undergraduates (11 male, *M_age_* = 20.14, *SD* = 1.91) took part in the research. Four participants (one male) failed to follow the instructions by responding with invalid key presses, thus were excluded from the analysis. All participants had normal or corrected-to-normal visual acuity. Informed consent was obtained from participants prior to the commencement of the experiment and the protocol was reviewed and approved by the Ethics Committee at the School of Psychology, University of Aberdeen, Scotland. The experiment had 2 (Expectancy: self-expected vs. friend-expected) × 2 (Owner: self vs. friend) repeated-measures design.

#### Stimulus materials and procedure

Participants arrived at the laboratory individually, were greeted by the experimenter, seated in front of a desktop computer, and informed that the experiment comprised an object-classification task featuring two categories of objects: pencils and pens. The task closely followed Experiment 2, but with two modifications. First, as stimulus-prioritisation was not observed when self-owned and friend-owned objects were equally likely to be presented during the task (see Experiments 1 and 2), this condition was dropped. Second, across two blocks of trials, although participants expected either self-objects or friend-objects to predominate (e.g., Block 1, 75% self-objects and 25% friend-objects; Block 2, 75% friend-objects and 25% self-objects), in reality the opposite was the case (e.g., Block 1, 25% self-objects and 75% friend-objects; Block 2, 25% friend-objects and 75% self-objects). The order of the experimental blocks was counterbalanced across participants and each block comprised 224 trials (e.g., 168 self-trials and 56 friend-trials). On completion of the task, participants were debriefed, thanked, and dismissed.

### Results

#### RT and accuracy

**Figure 3.**
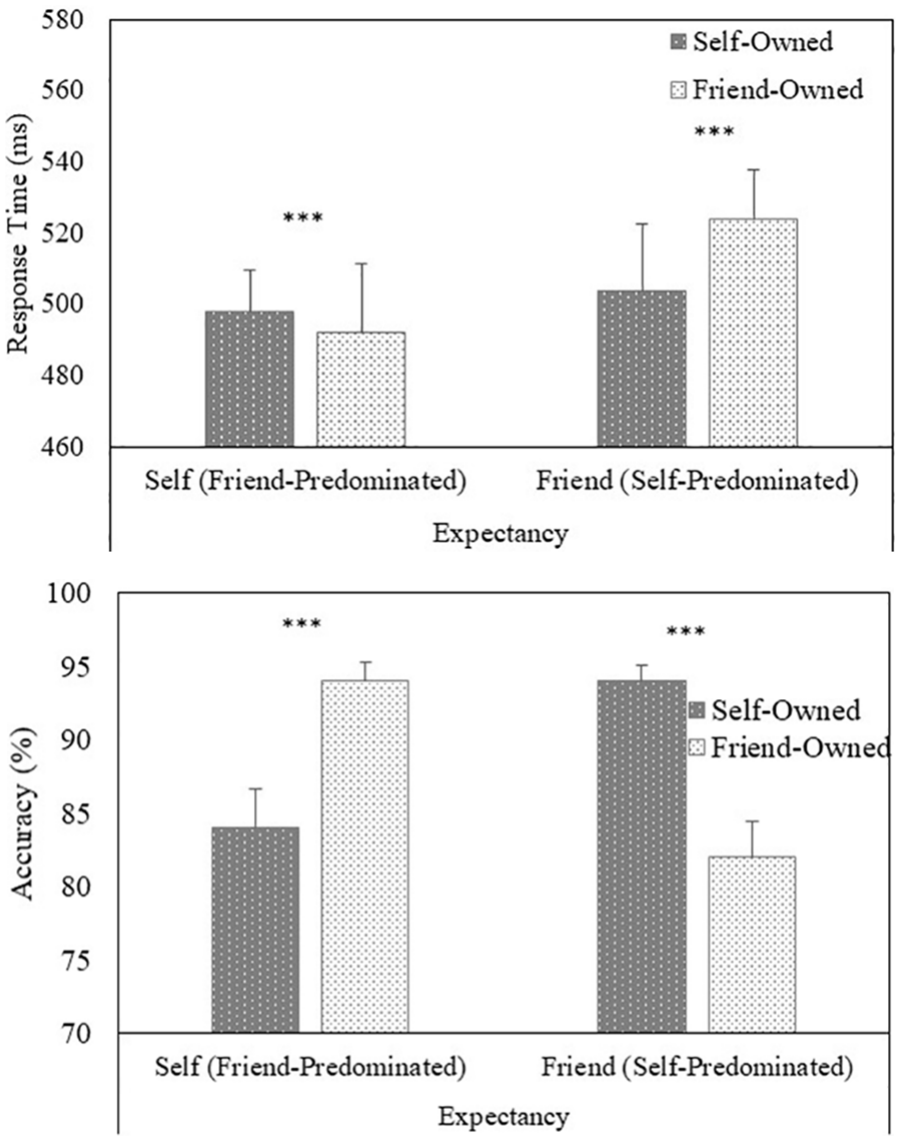
Task performance (upper panel = response time, lower panel = accuracy) as a function of Expectancy and Target (Experiment 3). Error bars represent +1 standard error of the mean Significant differences between conditions are depicted with asterisks: ***p < .001.

Responses faster than 200 ms were excluded from the analysis, eliminating approximately 2% of the overall number of trials. A multilevel model analysis of the RTs (correct responses) yielded a main effect of Expectancy (*b* = –.008, *SE* = .002, *t* = –4.94, *p* < .001), such that responses were faster in the self-expected (but friend-predominated) condition (*M* = 495 ms, *SD* = 88 ms) than the friend-expected (but self-predominated) condition (*M* = 514 ms, *SD* = 92 ms). A main effect of Owner (*b* = –.003, *SE* = .002, *t* = –2.03, *p* = .042) was also observed, indicating that RTs were faster to self-owned (*M* = 501 ms, *SD* = 86 ms) compared with friend-owned (*M* = 508 ms, *SD* = 94 ms) items. Finally, a significant Expectancy × Owner (*b* = .019, *SE* = .002, *t* = 10.95, *p* < .001) interaction emerged (see Figure 3). Further analysis of the interaction revealed that, when self-trials were expected (but friend-trials predominated), responses were faster to friend-owned compared with self-owned objects (*b* = .014, *SE* = .003, *t* = 6.07, *p* < .001). In contrast, when friend-trials were expected (but self-trials predominated), responses were faster to self-owned than to friend-owned objects (*b* = –.021, *SE* = .002, *t* = –8.44, *p* < .001).

A multilevel logistic regression analysis on the accuracy of responses reveal a significant Expectancy × Owner (*b* = –.629, *SE* = .032, *z* = –19.95, *p* < .001) interaction (see Figure 3). Further analysis of the interaction revealed that, when self-trials were expected (but friend-trials predominated), responses were more accurate to friend-owned compared with self-owned objects (*b* = –.598, *SE* = .045, *z* = –13.23, *p* < .001). In contrast, when friend-trials were expected (but self-trials predominated), responses were more accurate to self-owned than to friend-owned objects (*b* = .671, *SE* = .044, *t* = 15.16, *p* < .001).

#### Drift diffusion modelling

Following Experiments 1 and 2, seven models in which combinations of drift rate (*v*) varying as a function of Expectancy (i.e., self-expected vs. friend-expected) and Target (i.e., owned-by-self vs. owned-by-friend), and starting point (*z*) and threshold separation (*a*) varying as a function of Expectancy were estimated for comparison. As can be seen from Table 5, Model 4 yielded the best fit (i.e., lowest DIC value). To further evaluate this model, a PPC was also performed (Wiecki et al., 2013). This revealed good model fit (see Supplementary Material for associated plots).

**Table 5.** DIC for each model (Experiment 3).

Model	Expectancy	Owner	Fixed	DIC
1	*v*	*v*	*a, z*	−8,076
2	*z*	–	*a, v*	−11,435
3	*a*	–	*v, z*	−9,950
4	*a, z*	–	*v*	−21,570
5	*v, z*	*v*	*a*	−14,749
6	*a, v*	*v*	*z*	−11,031
7	*a, v, z*	*v*	–	−12,188

DIC: deviance information criterion; *v*: drift rate; *a*: threshold separation; *z*: starting point.

Inspection of the posterior distributions for the best fitting model revealed that task performance was underpinned by a response bias (see Table 6). When self-trials were expected but friend-trials predominated, comparison of the observed starting value (*z* = 0.40) with no bias (*z* = 0.50) revealed extremely strong evidence for a response bias, *p*_Bayes_(bias < .50) < .001, such that less information was needed to generate owned-by-friend compared with owned-by-self responses. Similarly, strong evidence for a response bias also emerged when friend-trials were expected but self-trials predominated (*z* = 0.62), indicating that less information was required when generating owned-by-self than owned-by-friend responses, *p*_Bayes_(bias > .50) < .001. No differences in threshold separation were observed.

**Table 6.** Parameter means and the upper (97.5q) and lower (2.5q) quantiles of the best fitting model (Experiment 3).

Diffusion model parameter	Mean	Quantile	
		2.5q	97.5q
*a_self-expected_*	1.213	1.161	1.397
*a_friend-expected_*	1.275	1.096	1.321
*v*	3.031	2.680	3.383
*z_self-expected_*	0.396	0.366	0.427
*z_friend-expected_*	0.622	0.591	0.652
*t*_0_	0.322	0.306	0.337
*sv*	1.065	0.961	1.170
*sz*	0.446	0.446	0.446
*st*	0.208	0.208	0.208

*a*: threshold separation; *v*: drift rate; *z*: starting point; *t*_0_: non-decision time; *sv*: inter-trial variability in drift rate; *sz*: inter-trial variability in starting point; *st*: inter-trial variability in non-decision time.

These findings develop and extend the effects observed in Experiment 2. Regardless of the prior expectancy that was in place (i.e., expect self-owned objects or expect friend-owned objects), stimulus prioritisation was driven by the objects that predominated during the task, resulting in the generation of both self- and friend-ownership effects. This reveals that participants were sensitive to discrepancies between their inaccurate prior beliefs and the prevalence of self-owned and friend-owned objects during the task. Corroborating Experiments 2 and 3, these ownership effects were underpinned by differences in the evidential requirements of response generation, such that less information was needed when responding to frequent compared with infrequent objects (De Loof et al., 2016; Domenech & Dreher, 2010; Dunovan et al., 2014; Mulder et al., 2012; White & Poldrack, 2014). These findings demonstrate the flexibility of ownership effects in task contexts in which prior beliefs are disconfirmed by ongoing sensory experiences (O’Callaghan et al., 2017; Otten et al., 2017).

## General discussion

A rapidly expanding literature has demonstrated the effects of self-relevance on decision-making (Humphreys & Sui, 2016; Sui & Humphreys, 2015, 2017; Sui & Rotshtein, 2019). Compared with stimuli paired with other social targets, those associated with the self are privileged during decisional processing, a prioritisation effect that is argued to be restricted to self-relevant material. Challenging this assumption, using an object-ownership paradigm, here we showed that stimulus prioritisation was sensitive to prior expectations about the prevalence of forthcoming items (owned-by-self vs. owned-by-friend) and whether these beliefs were supported during the task. Under conditions of stimulus uncertainty (i.e., no prior beliefs), replicating previous research, objects were classified more rapidly when owned-by-self compared with owned-by-friend (Golubickis et al., 2018, 2019). When, however, the frequency of stimulus presentation either confirmed or disconfirmed prior expectations, ownership effects were observed for both self-owned and friend-owned objects. These effects, moreover, were underpinned by a common underlying mechanism—the evidential requirements of response generation (White & Poldrack, 2014). Regardless of their owner, participants required less information when responding to probable compared with improbable stimuli (De Loof et al., 2016; Dunovan et al., 2014). These findings establish that, at least in the context of an object-ownership task, stimulus prioritisation effects can be generated for targets other than the self.

## The anatomy of self-prioritisation

Inspection of the extant literature on self-prioritisation yields an interesting observation. The apparent inevitability and exclusivity of the self-prioritisation effect derives, for the most part, from studies that have used either Sui et al.’s (2012) original shape-matching task or variants of this paradigm (Frings & Wentura, 2014; Mattan et al., 2015; Payne et al., 2017; Schäfer et al., 2015, 2016; Wozniak & Knoblich, 2019). Indeed, in other experimental contexts, self-prioritisation has proved considerably less reliable (Falbén et al., 2019; Siebold et al., 2015; Stein et al., 2016; Wade & Vickery, 2018). For example, using breaking continuous flash suppression (b-CFS) to explore the ease with which stimuli (i.e., Gabors) access visual awareness, Stein et al. (2016) reported no effect of self-relevance on the time taken for objects to overcome interocular suppression (cf. Macrae et al., 2017). Interestingly, however, a standard self-prioritisation effect was observed in a prior Gabor-label matching task. Similarly, Siebold et al. (2015) found no evidence that self-relevance enhanced the detection of stimuli (i.e., tilted lines associated with the self and a stranger) in a rapid oculomotor search paradigm, although again a self-prioritisation effect emerged in an earlier line-label matching task. Collectively these findings contest the assertion that, beyond explicit stimulus-label matching tasks, self-prioritisation is an obligatory effect (Sui & Humphreys, 2017). Rather than representing a mandatory facet of social cognition, self-prioritisation has the characteristics of a task-dependent phenomenon (Caughey et al., in press).

Work exploring the self-ownership effect also highlights the conditional automaticity of self-prioritisation (Constable et al., 2019; Falbén et al., 2019). As noted previously, when required to report which of two objects initially appeared on the computer screen (i.e., temporal order judgement task)—a mug owned-by-self or a mug owned-by-the-experimenter—participants were biased towards reporting that self-owned items appeared first (Constable et al., 2019; Experiment 1). This effect was eliminated, however, when the requested judgement was orthogonal to the dimension of interest (i.e., ownership). That is, when participants were asked to report whether a mug appeared to the left or right of fixation, self-prioritisation was abolished (Experiment 3; but see Truong et al., 2017). Corroborating this finding, in an object-classification task, Falbén et al. (2019) demonstrated that self-relevance only facilitated performance when task sets (e.g., reporting the ownership or identity of stimuli) directed attention to previously formed target–object associations (Hommel, 2004). When emphasis switched instead to a perceptual appraisal of stimuli, self-prioritisation failed to emerge (Siebold et al., 2015; Stein et al., 2016). At a minimum, therefore, self-prioritisation appears to necessitate task sets that facilitate access to target–object relations in memory (Kiefer, 2007; Maxfield, 1997).

Extending previous research on this topic, at least in the context of ownership, the current findings challenge the putative exclusivity of self-prioritisation during object processing (Golubickis et al., 2018, 2019). Confirming that previous demonstrations of the self-ownership effect were driven by an egocentric response-related strategy, but only under conditions of stimulus uncertainty (i.e., Experiment 1), here we provided evidence for the emergence of both self- and other-ownership effects when either accurate (i.e., Experiment 2) or inaccurate (i.e., Experiment 3) prior beliefs were provided. Interestingly, the manner in which stimulus-related expectancies were manipulated was likely crucial to the emergence of this effect. Previously, using a sequential version of the shape–label matching task, Sui et al. (2014) demonstrated that self-prioritisation was unaffected by the probability of stimulus presentation. That is, self-prioritisation emerged even when self-relevant (vs. other-relevant) stimuli appeared on only a minority of shape–label matching trials. It should be noted, however, that Sui et al. (2014) did not manipulate prior beliefs about the likelihood of stimulus presentation during the shape–label matching task. As such, the insensitivity of self-prioritisation to inter-trial stimulus contingencies does not speak to the effect that prior beliefs exert on task performance. For example, on a block-by-block or trial-by-trial basis (De Loof et al., 2016; Dunovan et al., 2014), prior information about the likelihood that self-relevant (or other-relevant) stimuli will be presented may wield considerable influence during a shape–label matching task (Sui et al., 2014).

## The origins of self-prioritisation

According to influential theoretical accounts, self-prioritisation is a perceptual phenomenon (Humphreys & Sui, 2016; Sui & Humphreys, 2015). Through reciprocal connections between regions of the prefrontal ventro-medial pre-frontal cortex (vMPFC) and temporal (posterior superior temporal sulcus (STS)) cortices, a Self-Attention Network (SAN) enhances the visual salience of self-relevant (vs. other-relevant) stimuli, thus triggering a self-prioritisation effect (but see Schäfer & Frings, 2019). As it turns out, however, evidence for this viewpoint is limited (e.g., Constable et al., 2019; Golubickis et al., 2017; Janczyk et al., 2019; Macrae et al., 2018; Reuther & Chakravarthi, 2017; Siebold et al., 2015; Stein et al., 2016). Indeed, disputing the perceptual account, recent research suggests that, during shape–label matching tasks, self-prioritisation emerges during a capacity-limited stage of central processing, thereby pinpointing short-term memory operations as a potential source of this effect (Janczyk et al., 2019; Reuther & Chakravarthi, 2017). Ownership tasks similarly fail to yield evidence that self-prioritisation originates in variation in the efficiency of perceptual processing. Rather, differences in the evidential requirements of response generation underpin the self-ownership effect (Golubickis et al., 2018, 2019). Replicating this finding, here we also showed that participants required less information when generating owned-by-self compared with owned-by-other responses. Critically, however, an equivalent response bias also underpinned the emergence of a friend-prioritisation effect, thereby furnishing further evidence for the post-perceptual origin of ownership effects.

In the current experimental context, stimulus prioritisation was sensitive to the extent to which prior beliefs about the prevalence of to-be-judged items were confirmed or disconfirmed by the frequency of object presentation during the task. Thus, consistent with a Bayesian account of decision-making—whereby predictions are updated in accordance with new information (Bar, 2007; Chater & Oaksford, 2008; O’Callaghan et al., 2017; Otten et al., 2017)—task performance was affected by the combination of prior knowledge and trial-by-trial sensory experiences. These effects, moreover, emerged whether objects were self-owned or belonged to a friend (Moutoussis et al., 2014). Notwithstanding the absence of self–other differences in stimulus prioritisation, such effects may nevertheless exist. Of importance may be the manner in which bias is defined and assessed. Take, for example, the stability of decisional biases. In Experiment 3, inaccurate prior beliefs were overridden by the frequency of stimulus presentation, whether the to-be-judged items were owned-by-self or owned-by-friend. It is possible, however, that the rate at which priors are updated may vary as a function of target (i.e., self vs. other), reflecting the status of the self as a fundamental information-processing hub (Humphreys & Sui, 2016; Sui & Humphreys, 2015, 2017; Sui & Rotshtein, 2019). In addition, updating may be sensitive to specific properties of objects—including valence and value—that have implications for the self-concept (e.g., self-enhancement motivation; Golubickis, Ho et al., in press; Sedikides & Gregg, 2008) or people’s evaluations of others. Using appropriate methodological/analytical techniques, a useful task for future research will be to explore these issues.

To develop a comprehensive understanding of item prioritisation (both self and other), additional research should probe the effects of object relevance in subtle and indirect ways using diverse tasks, stimuli, and measures (Constable et al., 2019; Falbén et al., 2019; Macrae et al., 2018; Siebold et al., 2015; Stein et al., 2016; Sui et al., 2012; Truong et al., 2017). It remains to be seen whether the current effects would extend to paradigms in which the stimuli have greater meaning for people and self-object relations are created and assessed in different ways (e.g., perceptual-matching tasks, prior-entry effects, visual search). For example, it is possible that, in combination with a response bias, prioritisation effects may be underpinned by differences in the efficiency of visual processing (i.e., drift rates) when stimuli have particular significance for people or the difficulty of the task is increased (Golubickis, Ho et al., in press). In addition, although the current findings demonstrate that self-prioritisation can be eliminated by prior knowledge indicating the prevalence of to-be-judged stimuli, whether this is consistently the case remains an open question. That is, whether self-relevance or stimulus probability is used as a heuristic to drive information processing likely reflects the influence of the specific task context, the items under consideration, and people’s prevailing goal states.

In extending the current line of inquiry, a less rigid characterisation of the self-concept should be adopted. To date, work in this area has tended to portray the self as a monolithic cognitive structure (Humphreys & Sui, 2016; Sui & Gu, 2017; Sui & Humphreys, 2017), a formerly commonplace but now outdated theoretical viewpoint. Rather than comprising a unitary representation, the self-concept is a dynamic, multifaceted construct, shaped by the interplay of cultural forces, pre-existing knowledge, situational factors, and transient processing concerns (Conway & Pleydell-Pearce, 2000; McConnell, 2011; Oyserman, 2007). This nuanced conception has obvious implications for stimulus prioritisation. Just as processing is sensitive to between-target differences (Sui et al., 2012, 2014), so too it may be responsive to the significance or meaning that stimuli hold for individuals given the specific aspect of the self-concept (or friend-concept) that is active at any given moment (i.e., within-person differences, see Golubickis et al., 2017, 2020; Macrae et al., 2018). Operating in this way, identity-based processing provides the flexibility that optimal social-cognitive functioning demands. Of interest, therefore, would be work exploring how prior beliefs about identity-relevant components of the self-concept (or friend-concept) together with ongoing sensory experiences influence stimulus prioritisation.

## Conclusion

Challenging the assumption that stimulus prioritisation is restricted to self-relevant material (Golubickis et al., 2018, 2019; Humphreys & Sui, 2016; Sui & Humphreys, 2015, 2017), here we demonstrated that prior beliefs in combination with stimulus prevalence moderate the emergence of both self- and other-ownership effects. Furthermore, these effects were underpinned by differences in the evidential requirements of response generation, such that less information was needed when responding to likely (vs. unlikely) objects, regardless of their owner (De Loof et al., 2016; Dunovan et al., 2014). Whether the non-exclusivity of self-prioritisation extends to other task contexts, however, has yet to be established.

## Supplemental Material

QJE-STD-19-458.R1-Supplementary_Materials – Supplemental material for It’s not always about me: The effects of prior beliefs and stimulus prevalence on self–other prioritisation Click here for additional data file. Supplemental material, QJE-STD-19-458.R1-Supplementary_Materials for It’s not always about me: The effects of prior beliefs and stimulus prevalence on self–other prioritisation by Johanna K Falbén, Marius Golubickis, Darja Wischerath, Dimitra Tsamadi, Linn M Persson, Siobhan Caughey, Saga L Svensson and C Neil Macrae in Quarterly Journal of Experimental Psychology

## Notes

1. Based on Golubickis et al. (2018), G*Power (*f* = .29, α = .05, power = 80%) revealed a requirement of 72 participants to detect the hypothesised interaction (Experiment 1). For subsequent repeated-measures designs (i.e., Experiments 2 and 3), 36 participants were recruited.

2. Bayesian *p* values quantify the degree to which the difference in the posterior distribution is consistent with the hypothesis. For example, a Bayesian *p* of .05 indicates that 95% of the posterior distribution supports the hypothesis.
