# Supplementary material for: It’s not always about me: The effects of prior beliefs and stimulus prevalence on self–other prioritisation
Source: Q J Exp Psychol (Hove). 2020 Apr 15;73(9):1466–80. doi: 10.1177/1747021820913016 (PMC7604934; doi:10.1177/1747021820913016)
Supplement: QJE-STD-19-458.R1-Supplementary_Materials – Supplemental material for It’s not always about me: The effects of prior beliefs and stimulus prevalence on self–other prioritisation [file QJE-STD-19-458.R1-Supplementary_Materials.docx]

Supplementary Material for:

**It’s Not Always About Me:**

**The Effects of Prior Beliefs and Stimulus Prevalence on Self-Other Prioritization**

Johanna K. Falbén, Marius Golubickis, Darja Wischerath, Dimitra Tsamadi,

Linn M. Persson, Siobhan Caughey, Saga L. Svensson, C. Neil Macrae


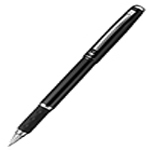

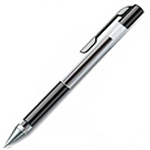

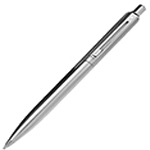


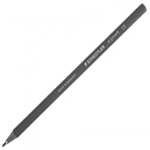

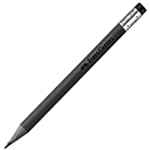

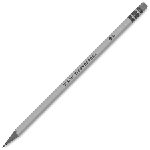


*Figure S1*. Examples of the experimental stimuli (i.e., pens and pencils).


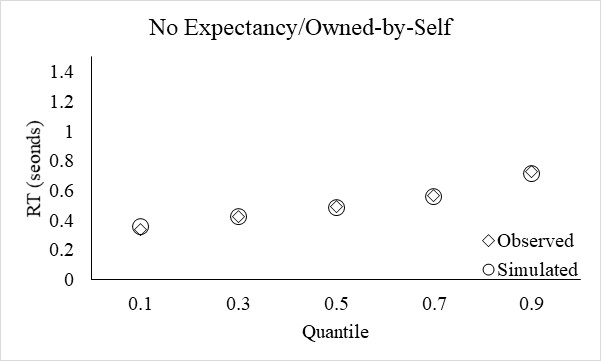

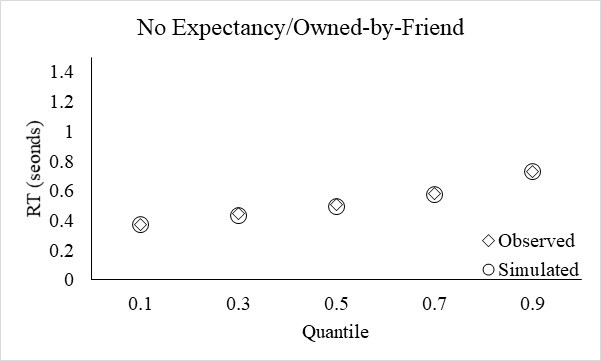

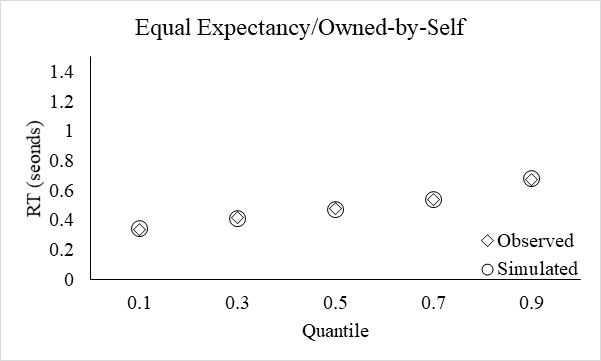

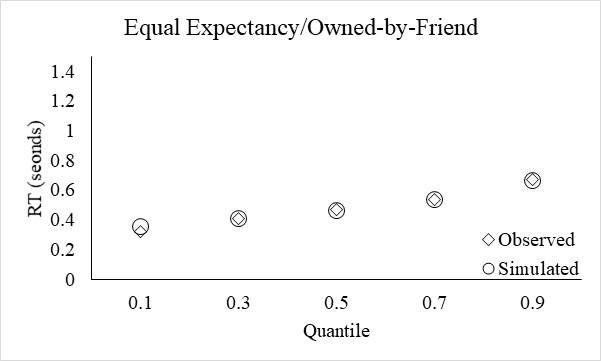


*Figure* *S2*. Comparison of simulated data generated by the best fitting model (i.e., model 7) and the observed data for each experimental condition for the .1, .3, .5, .7, and .9 RT quantiles (Experiment 1).


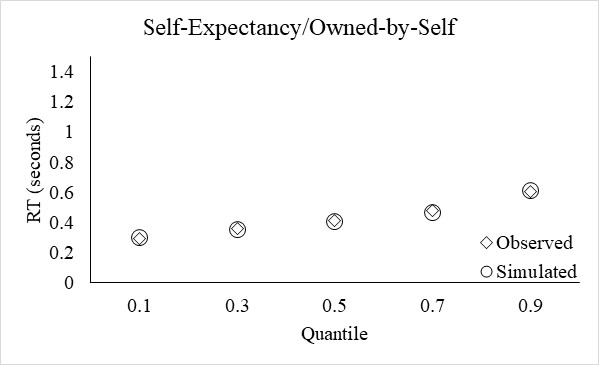

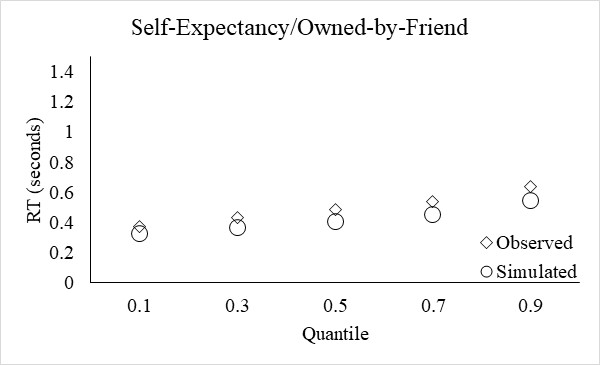

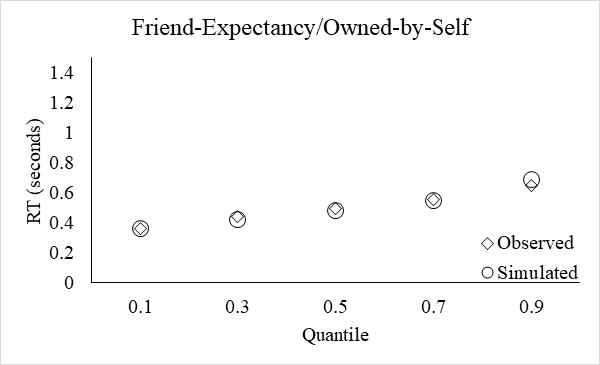

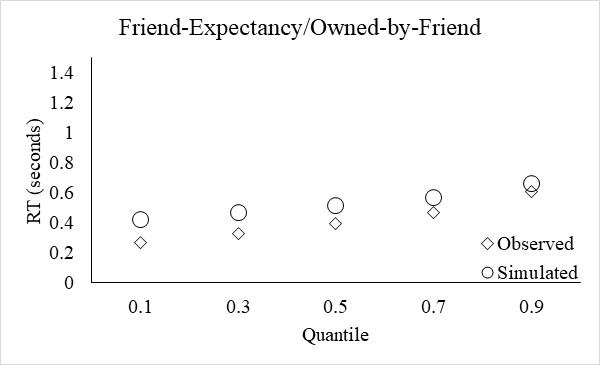

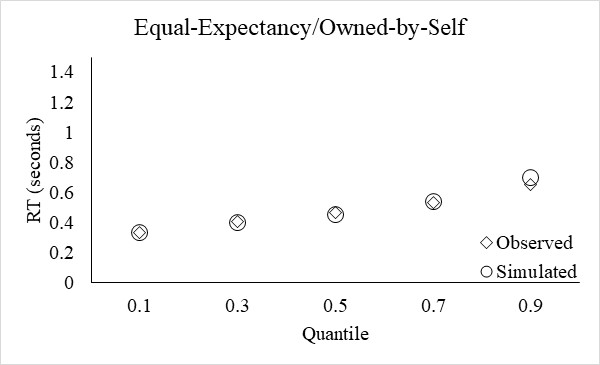

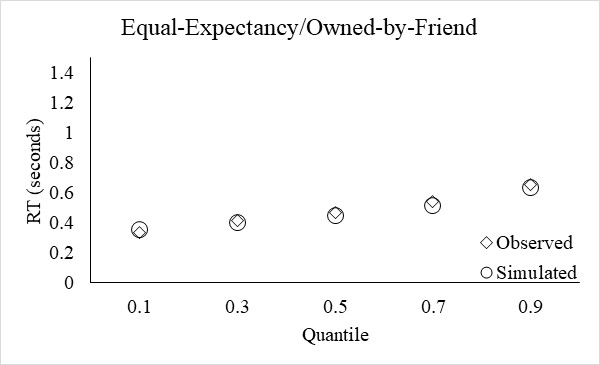


*Figure* *S3*. Comparison of simulated data generated by the best fitting model (i.e., model 7) and the observed data for each experimental condition for the .1, .3, .5, .7, and .9 RT quantiles (Experiment 2).

*
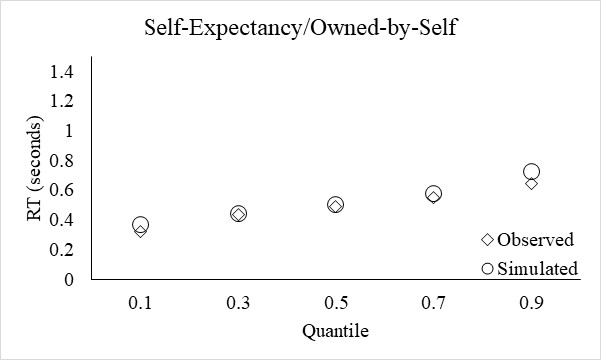

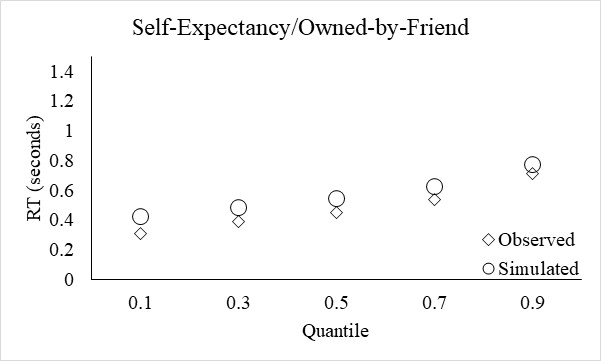

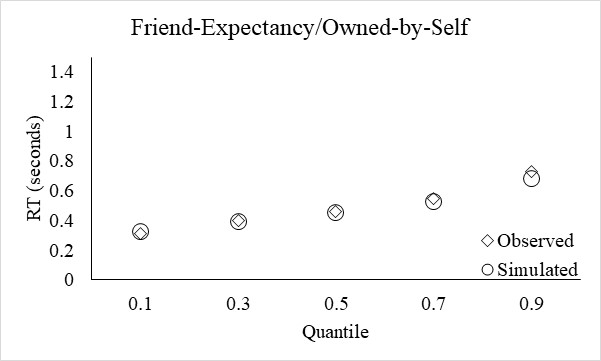

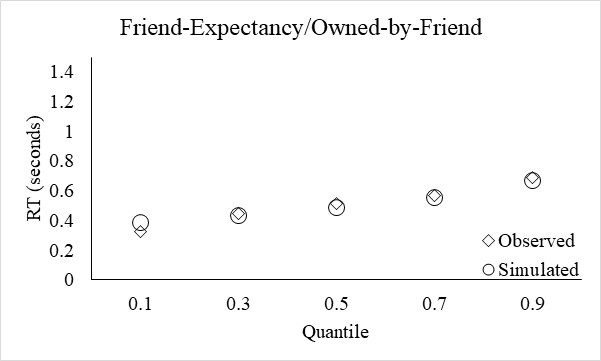
*

*Figure* *S4*. Comparison of simulated data generated by the best fitting model (i.e., model 4) and the observed data for each experimental condition for the .1, .3, .5, .7, and .9 RT quantiles (Experiment 3).


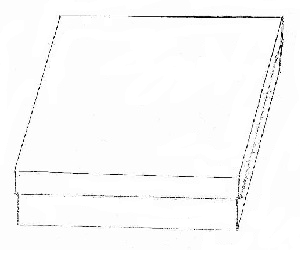

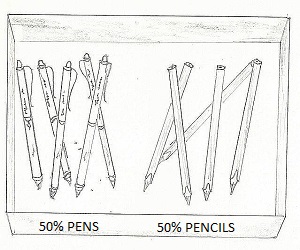


*Figure S5*. Examples of the pencil sketches of boxes containing pencils and pens (which presented indicative information about the trial structure during the task) from Experiment 1 (closed box = no expectancy, open box = 50/50 expectancy).


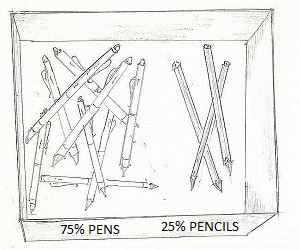

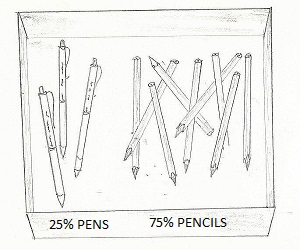

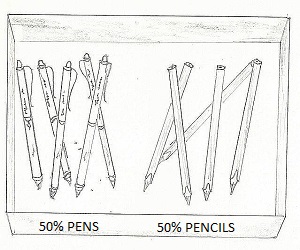


*Figure S6*. Examples of the pencil sketches of boxes containing pencils and pens (which presented indicative information about the trial structure during the task) from Experiment 2 (Self-expectancy [75% owned-by-self vs. 25% owned-by-friend], Friend-expectancy [75% owned-by-friend vs. 25% owned-by-self] and Equal-expectancy [50% owned-by-self vs. 50% owned-by-friend]).


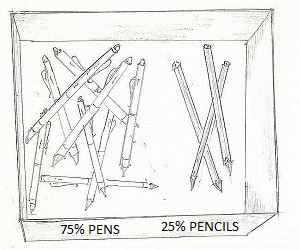

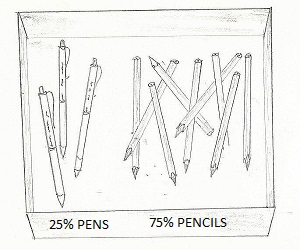


*Figure S7*. Examples of the pencil sketches of boxes containing pencils and pens (which presented indicative information about the trial structure during the task) from Experiment 3 (Self-expectancy [75% owned-by-self vs. 25% owned-by-friend], Friend-expectancy [75% owned-by-friend vs. 25% owned-by-self]).
